# The choroid plexus response to a repeated peripheral inflammatory stimulus

**DOI:** 10.1186/1471-2202-10-135

**Published:** 2009-11-18

**Authors:** Fernanda Marques, João C Sousa, Giovanni Coppola, Daniel H Geschwind, Nuno Sousa, Joana A Palha, Margarida Correia-Neves

**Affiliations:** 1 Life and Health Sciences Research Institute (ICVS), School of Health Sciences, University of Minho, Campus Gualtar, 4710-057 Braga, Portugal; 2 Program in Neurogenetics, Department of Neurology, David Geffen School of Medicine-UCLA, Los Angeles, USA

## Abstract

**Background:**

Chronic systemic inflammation triggers alterations in the central nervous system that may relate to the underlying inflammatory component reported in neurodegenerative disorders such as multiple sclerosis and Alzheimer's disease. However, it is far from being understood whether and how peripheral inflammation contributes to induce brain inflammatory response in such illnesses. As part of the barriers that separate the blood from the brain, the choroid plexus conveys inflammatory immune signals into the brain, largely through alterations in the composition of the cerebrospinal fluid.

**Results:**

In the present study we investigated the mouse choroid plexus gene expression profile, using microarray analyses, in response to a repeated inflammatory stimulus induced by the intraperitoneal administration of lipopolysaccharide every two weeks for a period of three months; mice were sacrificed 3 and 15 days after the last lipopolysaccharide injection. The data show that the choroid plexus displays a sustained response to the repeated inflammatory stimuli by altering the expression profile of several genes. From a total of 24,000 probes, 369 are up-regulated and 167 are down-regulated 3 days after the last lipopolysaccharide injection, while at 15 days the number decreases to 98 and 128, respectively. The pathways displaying the most significant changes include those facilitating entry of cells into the cerebrospinal fluid, and those participating in the innate immune response to infection.

**Conclusion:**

These observations contribute to a better understanding of the brain response to peripheral inflammation and pave the way to study their impact on the progression of several disorders of the central nervous system in which inflammation is known to be implicated.

## Background

Inflammation is implicated in the appearance and in the progression of central nervous system (CNS) diseases such as multiple sclerosis (MS) and Alzheimer's diseases (AD), although the mechanism underlying such involvement is poorly understood [1-4]. It is recognized that the inflammation observed in the CNS of subjects with some of these diseases may originate in the periphery [5-7], particularly when the inflammatory stimulus is persistent. Persistence may be due to chronic inflammation or to repeated exposure to acute inflammatory stimulus for long periods of time. Of relevance, persistent or chronic inflammatory signals result in excessive microglia activation and cause localized or disseminated tissue dysfunction and damage [8], ultimately resulting in accentuation of brain pathology.

The blood-brain barriers, constituted by the endothelial cells of the blood capillaries (blood-brain barrier-BBB) and by the epithelial cells of the choroid plexus (CP) that separate the blood from the cerebrospinal fluid (CSF), are key players in the communication between the periphery and the brain. However, most studies published to date address the BBB interactions in response to acute peripheral stimulus or in the context of CNS diseases [9,10]. Recently, we showed that the blood-CSF barrier is also an important mediator of acute peripheral inflammation into the CNS [11]. Of notice, this response triggers molecular pathways that are commonly viewed as both neuroprotective and deleterious for the brain. Whilst the secretion of proinflammatory cytokines into the CSF, the decreased expression of proteins that form the epithelial cells tight junctions and the increased expression of proteins that may facilitate leukocyte trafficking into the brain might be predicted to display deleterious effects, the response can be also consider protective since it modulates iron metabolism in a way that may prevent microorganism replication in the CSF and, consequently, dissemination within the brain [12,13]. While these observations associate the CP response to acute peripheral inflammation, they raise the possibility that the CP may as well be equipped to mount a sustained response to persistent peripheral inflammatory stimuli.

Importantly, scattered but relevant reports have shown that the CP may contribute to the aetiology of CNS diseases in which persistent, rather than acute inflammation, is more likely to trigger CNS disease. In MS, and in animal models of MS, the CP was proposed as the main route of leukocyte entry into the brain [14,15]. In AD, it was proposed that the CP participates in amyloid β peptide clearance out of the brain through CSF carrier proteins (e.g. transthyretin and apolipoprotein J) [16] that bind to receptors (e.g. megalin) [17] in the apical membrane of the CP epithelial cells.

These observations prompted us to investigate, using microarray analysis, how the CP transmits immune signals into the brain in response to peripheral repetitive inflammation. We found that the CP displays a low intensity, but sustained, response to this stimulus and that the leukocyte extravasation signalling pathway as well as pathways that mediate the innate immune response to infection are the most altered both 3 and 15 days after the last LPS injection.

## Results

### Repeated peripheral inflammation induces alterations in the choroid plexus transcriptome

Mice were administered LPS once every 2 weeks for 3 months and were sacrificed 3 and 15 days after the last LPS injection. The first time-point for sacrifice was chosen taking into account previous reports [11] showing that 3 days after a single LPS injection few genes display an altered expression. Comparing the gene expression profile 3 days after the last LPS injection in the present protocol with that occurring 3 days after a single LPS administration, allowed us to identify the genes whose altered expression is the result of the repeated rather than single exposure to the inflammatory stimuli. Since in the present experimental protocol LPS was administered every 2 weeks for 3 months, the analysis 15 days after the last injection allowed us to evaluate if any changes occurring in the gene expression profile were sustained during repeated exposure to inflammatory stimuli.

During the duration of the experiment, the LPS dose used did not induce any statistical significant changes in the body weight, and the survival rate observed was of 100%. No cage behaviour alterations were observed throughout the experimental period.

**Figure 1:**
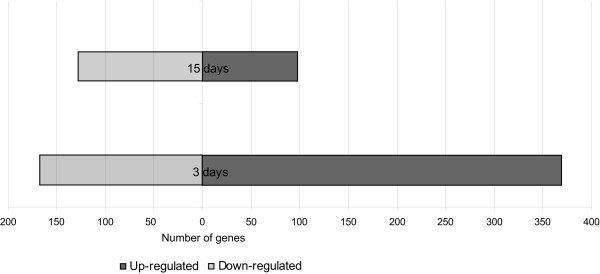
**Chronic inflammation alters the choroid plexus gene expression profile**. Number of genes up-regulated (black) and down-regulated (gray) in the choroid plexus 3 and 15 days after the last LPS injection. All genes with a variation in expression of at least 10% (FDR 5%) were considered.

Figure 1 shows that 3 days after the last LPS injection 536 probes have an altered expression when compared to the saline injected animals. Since at the same time-point after a single LPS injection [11], only 46 genes were found to have an altered expression, this response depends on the chronic nature of the stimuli. Moreover, a response of the CP to the continuous peripheral stimuli is still present 15 days after the last injection, with the expression of 226 genes altered. This suggests that, contrary to what is observed upon a single acute stimulus, repetitive LPS injections do not allow the CP gene expression profile to restore to its basal levels in a short period of time.

**Figure 2:**
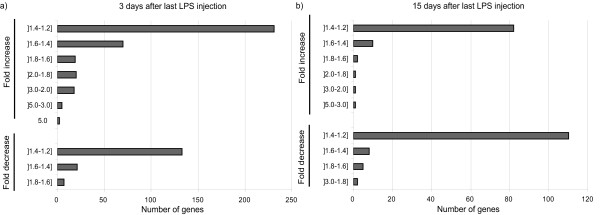
**Fold changes in gene expression**. The fold change induced in most genes by the chronic stimulus is below 50%, both at 3 days (a) and at 15 days (b) after the last LPS injection.

Despite the number of genes whose expression is found altered, not many display a fold change higher than 50% (Figure 2). In fact, as can be observed in Figure 2a, 3 days after the last LPS injection, only 25 genes out of the 369 genes whose expression is up-regulated display a fold change ≥ 2,0. In addition, from the 167 genes whose expression is down-modulated, none shows a strong down-modulation and only 5 display a decrease as low as 1,7 fold (Figure 2a). The fold changes are even smaller (but still statistically significant) at 15 days after the last LPS injection (Figure 2b); only 2 genes present a fold increase ≥ 2,0.

Table 1 lists, taking into consideration the fold change, the 10 genes whose expression is most altered at 3 and 15 days after the last LPS injection.

**Table 1:** List of genes whose expression is most altered at 3 and 15 days after last LPS injection.

3 days after last LPS injection				15 days after last LPS injection			
**Up-regulated**				**Up-regulated**			
**Transcript**	**Symbol**	**Definition**	**Fold change**	**Transcript**	**Symbol**	**Definition**	**Fold change**
NM_011315	Saa3	Serum amyloid A 3	12,1	NM_016974.1	Dbp	D site albumin promoter binding protein	3,3
NM_008161.1	Gpx3	Glutathione peroxidase 3	6,7	NM_145434.1	Nr1d1	Nuclear receptor subfamily 1 group D member 1	2,5
NM_017372.2	Lyzs	Lysozyme	4,2	NM_008176.1	Cxcl1	Chemokine (C-X-C motif) ligand 1	1,9
NM_009252.1	Serpina3n	Serine (or cysteine) proteinase inhibitor clade A member 3N	3,8	NM_017372.2	Lyzs	Lysozyme	1,7
NM_008134.1	Glycam1	Glycosylation dependent cell adhesion molecule 1	3,7	NM_011328.1	Sct	Secretin	1,6
NM_030707	Msr2	Macrophage scavenger receptor 2	3,2	NM_008161.1	Gpx3	Glutathione peroxidase 3	1,5
NM_013653.1	Ccl5	Chemokine (C-C motif) ligand 5	3,1	NM_175030.1	Tctex1d4	Tctex1 domain containing 4	1,5
NM_010185.2	Fcer1g	Fc receptor IgE high affinity I gamma polypeptide	2,9	NM_010493.2	Icam1	Intercellular adhesion molecule	1,5
NM_007574.1	C1qg	Complement component 1 q subcomponent gamma polypeptide	2,8	NM_009778.1	C3	Complement component 3	1,4
NM_008491.1	Lcn2	Lipocalin 2	2,7	NM_030707	Msr2	Macrophage scavenger receptor 2	1,4

**Down-regulated**				**Down-regulated**			
**Transcript**	**Symbol**	**Definition**	**Fold change**	**Transcript**	**Symbol**	**Definition**	**Fold change**
XM_355574.1	Gnai1	Guanine nucleotide binding protein alpha inhibiting 1	-1,7	NM_007489.1	Arntl	Aryl hydrocarbon receptor nuclear translocator-like	-2,8
NM_177644.2	Rasal2	RAS protein activator like 2	-1,7	NM_175475.2	Cyp26b1	Cytochrome P450 family 26 subfamily b polypeptide 1	-2,3
NM_018824.2	Slc23a2	Solute carrier family 23 (nucleobase transporters) member 2	-1,7	NM_133903.2	Spon2	Spondin 2 extracellular matrix protein	-1,7
NM_011638.3	Tfrc	Transferrin receptor	-1,7	NM_029720.1	Creld2	Cysteine-rich with EGF-like domains 2	-1,6
NM_178404.2	Zc3hdc6	Zinc finger CCCH type containing 6	-1,7	NM_175930.2	Rapgef5	Rap guanine nucleotide exchange factor (GEF) 5	-1,6
NM_013519.1	Foxc2	Forkhead box C2	-1,6	NM_198885.2	Scx	Scleraxis	-1,6
NM_148930.2	Rbm5	RNA binding motif protein 5	-1,6	NM_175930.2	Rapgef5	Rap guanine nucleotide exchange factor (GEF) 5	-1,6
NM_172310.1	Tarsl2	Threonyl-tRNA synthetase-like 2	-1,5	NM_207261.1	Kcnk18	Potassium channel, subfamily K, member 18	-1,5
NM_028021.1	Myh14	Myosin, heavy polypeptide 14	-1,5	NM_013559.1	Hsp105	Heat shock protein 105	-1,4
NM_009222.2	Snap23	Synaptosomal-associated protein 23	-1,5	NM_030143.2	Ddit4l	DNA-damage-inducible transcript 4-like	-1,4

To identify the genes whose altered expression was sustained we looked for those that had at least a 40% change, both at 3 and at 15 days after the last LPS stimulus. Only 7 up-regulated genes fulfil such criteria (Table 2).

**Table 2:** Genes whose expression is at least 40% up-regulated both at 3 and 15 days after the last LPS injection.

Genes altered 3 and 15 days after last LPS injection				
			**Fold change**	
			
**Transcript**	**Symbol**	**Definition**	**3 days**	**15 days**

NM_008161.1	Gpx3	Glutathione peroxidase 3	6,7	1,5
NM_017372.2	Lyzs	Lysozyme	4,2	1,7
NM_030707	Msr2	Macrophage scavenger receptor 2	3,2	1,4
NM_009778.1	C3	Complement component 3	2,6	1,4
NM_008176.1	Cxcl1	Chemokine (C-X-C motif) ligand 1	2,0	1,9
NM_011328.1	Sct	Secretin	1,6	1,6
NM_010493.2	Icam1	Intercellular adhesion molecule	1,5	1,5

### Identification of altered gene pathways

We next analysed, using the Ingenuity software, the pathways to which the genes with altered expression belong. From Table 3, it is clear that 15 days after the last LPS injection only a few genes within each biological pathway remained with altered expression. Interestingly, the signalling pathways in which a considerable number of genes still have altered expression 15 days after the last injection of LPS are those related with leukocyte migration and with the complement cascade signalling.

**Table 3:** Clustering of the genes whose expression is altered in the choroid plexus upon repeated peripheral LPS injection.

Atered genes			
		**3 days**	**15 days**

**Signaling pathways**	Toll like receptor signaling	Tlr7; Mapk8; Cd14; Relb ↑	-
	
	T cell receptor signaling	Mapk8; Nfat5;Btk; Vav1; Relb ↑; Rras2; Nfatc1 ↓	Itk ↑
	
	B cell receptor signaling	Fcgr2a; Bcl2a1; Mapk8; Nfat5; Map2k7; Rac2; Fcgr2b; Inpp5d; Btk; Vav1; Relb ↑; Rras2; Nfatc1; Bcl2l1 ↓	-
	
	SAPK JNK signaling	Gpr65; Gpr24; Mapk8; Map2k7; Rac2; Sh2d2a ↑; Rras2; Fadd; Nfatc1; Egfr ↓	Gng11;Gpr34 ↑; Dusp8 ↓
	
	Natural killer signaling	Fcer1g; Tyrobp; Siglec7; Rac2; Klrd1; Inpp5d; Vav1; Hcst ↑; Rras2 ↓	Klrd1; Siglec7 ↑
	
	NF-kB signaling	Tlr7; Tnfsf13b; Mapk8; Map2k7; Relb ↑; Rras2; Egf; Tnfsf11; Egfr ↓	Tnfsf13b ↑
	
	JAK/STAT signaling	Stat3 ↑; Rras2 ↓	-
	
	cAMP mediated signaling	Rgs10; Grm3; Stat3 ↑; Gnai; Grk4; Cngb1; Rgs12 ↓	Adcy9; Crem ↓
	
	Interferon signaling	Isgf3g; Tap1; Ifngr2; Ifitm1; Ifitm3; Ifi205; Irf5; Ifngr2 ↑	Ifitm1 ↑
	
	GM-CSF signaling	Bcl2a1; Stat3 ↑; Rras2; Bcl2l1 ↓	-
	
	Integrin signaling	Mapk8; Actin4; Rac2 ↑; Rras2; Egfr ↓	Itgae; Itgb7 ↑
	
	IL-10 signaling	Il10ra; Tcgr2a; Mapk8; Cd14; Fcgr2b; Stat3; Relb ↑	-
	
	IL-6 signaling	Mapk8; Map2k7; Cd14; Stat3; Relb ↑; Rras2 ↓	-
	
	IL-4 signaling	Nfat5; Il2rg; Inpp5d ↑; Rras2; Nfatc1 ↓	-
	
	IL-2 signaling	Mapk8; Il2rg ↑; Rras2 ↓	-
	
	Complement/coagulation cascade signaling	C3; C1qa; C1qb; C1qg; Vwf; Serping1; C2 ↑	Serpind1; C1qg; C1qa; C3 ↓
	
	Apoptosis signaling	Casp1; Bcl2a1; Mapk8; Map2k7; Relb ↑; Rras2; Bcl2l1; Ecfr; Bcl2 ↓	Card14 ↑
	
	Antigen presentation pathway	B2m; H2-T23; Tap1; Psmb9 ↑	-

**Acute phase response**	Acute phase proteins	Serping1; Map2k7; C3; Mapk8;Saa3;Vwf; Stat3; Slp; C2; serpina3n ↑; Fn1; Rras2 ↓	C3 ↑

**Leukocyte migration**	Leukocyte extravasation signaling	Ncf2; Selpl; Mapk8; Cyba; Jam2; Actn4; Rac2; Mmp23; Icam1; Glycam1; Madcam1; Timp1; Btk; Vav1 ↑; Gnai ↓	Icam1; Itk; Cyba ↑
	
	Extracellular matrix	Timp1; Dcn; Lamb2; Fbln1; Mmp23; Tecta; Emid1 ↑; Fn1 ↓	Tnc; Spon2 ↓
	
	Cytokine signaling	Il16; Ccl5; Cxcl1; Ccl7; Ccl13; Xcl1; Cxcl16; Ccl2 ↑; Gnai1; Rras2 ↓	Cxcl1; Ccl7; Xcl1; Ccl2 ↑
	
	Actin cytoskeleton signaling	Nckap1l; Gpr65; Gpr34; Actn4; Kng1; Rac2; Cd14; Vav1 ↑; Rras2; Fgf7; Egf; Myh14; Fn1; Egfr ↓	Gpr34 ↑
	
	Axonal guidance signaling	Igf1; Nfat5; Rac2; Fes ↑; Gnai1; Slit2; Rras2; Rgma; Nfatc1; Egf; Egfr ↓	Rgmb ↑

In a previous report we characterized the kinetic gene expression profile [from 1 h up to 3 days] in response to a single LPS peripheral injection [11]. When the transcriptome of the CP at 3 days after this acute inflammatory stimulus is compared to that observed 3 days after the last injection of the repeated protocol reported here, it should be noted that: only 4 genes (rather than 12) of the acute phase response signalling pathway, 2 genes (rather than 7) encoding for chemokines, 2 genes (rather than 5) of the complement system and 3 (rather than 4) from the antigen presentation pathway are common in both responses. All other pathways, such as that involved in axonal guidance signalling, are only implicated when repeated stimuli are imposed, suggesting that most of these alterations are certainly associated with the chronicity of the model reported here.

### Confirmation of array results by qRT-PCR on a set of relevant genes

**Figure 3:**
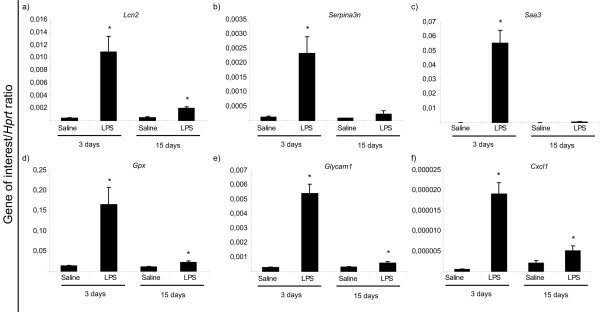
**qRT-PCR analysis of the expression of selected genes**. Confirming the array results, the expression of *Lcn2*, *Serpina3n*, *Saa3*, *Cxcl1*, *Gpx3*, and *Glycam1 *(a-f) was found up-regulated by qRT-PCR.

Within each pathway, and using RNA extracted from CP pools of an independent experiment, a number of genes found up-regulated (*Lcn2*, *Serpin3n*, *Saa3*, *Cxcl1*, *Gpx3 *and *Glycam1*) were chosen for qPCR analysis; this analysis confirmed the array data. Figure 3 exemplifies the expression levels of some of these genes. Relatively to the down-regulated genes although all the genes tested showed a decreased expression they did not reach statistical significance (data not shown); this could be due to the fact that all the down-regulated genes showed only a slightly decrease in the array (Figure 2).

## Discussion and conclusion

This study shows that sustained peripheral inflammation induced by repeated administration of LPS, every 2 weeks for 3 months, causes an altered CP transcriptome, 3 and 15 days after the last LPS injection. We have previously shown that an acute LPS injection triggers a rapid and transient alteration in the CP gene expression profile, that returns almost to basal levels after 3 days. In this study, we show that facing repeated LPS stimuli the number of genes whose expression is altered in the CP, compared with the number of genes altered 3 days after an acute LPS injection, is much higher. Another important difference is that when compared the present study with the overall CP response to acute LPS it is clear that the magnitude of the fold changes is now much lower. Therefore, we can conclude that the chronicity of the inflammatory stimuli alters the dynamics of the CP response. Indeed, it seems that the repeated injection of LPS induces, in the CP, a sustained transcription of specific genes encoding for molecules already found transiently altered upon a single LPS injection. These include molecules known to participate in the host response against microorganisms, elements of the complement and chemokines.

When the overall CP response is evaluated in terms of the major biological pathways, 3 days after the last LPS administration, the CP response is mainly characterized by the increased expression of genes encoding for chemokines, molecules of the complement, and molecules involved in leukocyte extravasation signalling and in the activation of NK, T and B cells. As expected, genes belonging to signalling transduction pathways are similarly altered and are known to mediate the regulation of genes encoding for molecules such as cytokines and molecules of the complement. Of notice, it is well established that a chronic inflammation can result in the inappropriate recruitment of leukocytes and cause localized or disseminated tissue dysfunction and damage. The "leukocyte extravasation signaling" pathway seems, in fact, the most altered both at 3 and 15 days after the last LPS treatment. This includes the increased expression of genes encoding for cell adhesion molecules such as ICAM-1, glycosylation dependent cell adhesion molecule 1 (*Glycam1*), mucosal vascular addressin cell adhesion molecule 1 (*Madcam1*), junction adhesion molecule 2 (*Jam2*) and selectin P ligand (*Selpl*); chemokines that are required for trafficking of immune cells from the blood into tissues such as *Xcl1*, *Ccl7*, *Cxcl1*, *Ccl2 *and interleukins such as interleukine-16 (IL-16). Of notice, we did not find IL-16 expression influenced by acute inflammation [11]. IL-16 is a pleiotropic cytokine that is a natural ligand of CD4 [18,19] and has been identified at sites of allergic inflammation in both the murine and the human airway epithelium [20,21]. This cytokine is known as a chemoattractant for CD4^+ ^T cells, monocytes, eosinophils and dendritic cells, with preferential chemoattractant activity for the CD4^+ ^Th1 subset [22-24]. Despite the increased expression of genes encoding for molecules that participate in leukocyte recruitment, no changes were observed in the expression levels of genes that encode tight junction proteins, neither an increase in the number of cells was observed in the CSF or gross morphological changes in the CP (data not shown). It is therefore important to further understand whether the repeated exposure to peripheral inflammation ultimately results in the entry of immune cells into the brain, or whether additional conditions are necessary for such to occur.

Another interesting finding is the effect of repeated exposure to LPS in the expression of genes that encode for proteins involved in axonal guidance. This suggests that the chronicity of the stimulus can induce alterations in the normal neuronal morphology and neuronal plasticity. One of such molecules whose gene expression is decreased is the slit homolog 2 (*Slit2*). Interestingly SLIT, a secreted protein known for its role of repulsion in axon guidance and neuronal migration [25,26], can also inhibit leukocyte chemotaxis induced by chemotactic factors [26]. In addition to *Slit2*, the expression of the gene encoding for the RGM domain family member A (*Rgma*) is decreased in the CP after sustained inflammation. RGMa is suggested to inhibit axon growth and synapse formation [27]. In normal brains, RGMa expression is detected on the perikarya of some neurons, CP, smooth muscle, endothelial cells, oligodendrocytes, and myelinated white matter fibers [28]. Interestingly, and probably also with impact on the brain parenchyma, is the altered expression of the gene encoding for secretin which is increased at both time points analysed, and one of the most altered 15 days after the last LPS injection. The secretin gene is known to be expressed in several developing brain regions namely by the CP [29]. The up regulation of its expression may be protective for the brain parenchyma in response to LPS since secretin deficient mice display impaired synaptic plasticity in the CA1 area of the hippocampus [30] and given the neuroprotective role secretin exerts on neuronal progenitor cells against ethanol-mediated neurotoxicity [31]. While some proteins secreted into the CSF may exert a function in the brain parenchyma, others may influence the CP in itself. Among these is glutathione peroxidase 3. By participating in the detoxification of reactive oxygen spices, which are formed during an inflammatory response, the increased expression of this antioxidant defence enzyme [32] can be protective to the CP epithelial cells.

Further comparison between the gene expression profiles after exposure to a single or to repeated LPS injections, shows that in chronicity the fold change is considerably smaller. However, it should be noted that the expression of some CP genes seems solely altered after the repeated stimulation. Among these are genes encoding for proteins of the S100 family. The S100 family of calcium-binding proteins comprises a new group of pro-inflammatory molecules that has been discussed in the context of MS [33] and of AD [34]. Here we show increased levels of S100a8 and S100a9. Another molecule exclusively induced during the chronic treatment is the macrophage scavenger receptor 2 (*Msr2*). Scavenger receptors (SRs), initially described on macrophages as high-affinity receptors for acetylated low-density lipoproteins, comprise several receptor classes [35,36] and are expressed in various cell types. SRs have a role in the binding and internalization of many unrelated ligands, such as fibrillar β-amyloid, lipids, glycated collagen and apoptotic cells and, therefore, are important for tissue homeostasis. The up-regulated expression of this gene could indicate a protective role of the CP in the progression of diseases such as AD. As referred above, clearance of amyloid β peptide out of the brain is reported to occur through the megalin and low-density lipoprotein receptor-related protein receptors that are SRs [37].

The CP is composed of a vascularized stroma surrounded by a tight layer of epithelial cells that are responsible for producing most of the CSF. Therefore any alteration in the CP gene expression profile may influence the CSF composition, which may then be transmitted to the brain parenchyma. While many of the genes whose expression we found altered in the present study are expressed in the CP epithelial cells [11,12], we cannot exclude the contribution of other cells of the CP stroma, such as endothelial cells and macrophages in the observed response.

In summary, we describe here that the CP displays a sustained response to a repeated inflammatory stimulus induced in the periphery. Importantly, this response seems to share some mechanisms previously described for the BBB, which include the activation of adhesion and chemoattraction signals in endothelial cells in CNS diseases such as MS [9,38]. Therefore, both the blood-CSF barrier and the BBB seem equipped to convey signals to the brain parenchyma in response to both acute and chronic inflammation. Future studies should further investigate the role of the CP response in the context of CNS disorders.

## Methods

### Animals and LPS injection

All experiments were conducted using 8-9 week-old C57BL/6 male mice (Charles River, Barcelona, Spain), in accordance with the European Community Council Directive 86/09/EEC guidelines for the care and handling of laboratory animals. Animals were maintained in 12 h light/dark cycles at 22-24°C and 55% humidity and fed with regular rodent's chow and tap water *ad libitum*. Animals were handled for 1 week prior to the beginning of the experiment, in order to reduce the stress induced by the injection. Animals were given LPS (*Escherichia coli*, serotype O26:B6; Sigma, St. Louis, USA) (5 μg/g body weight) intraperitoneally (*i.p*.); control animals were injected with vehicle (0.9% NaCl) alone. Mice received *i.p*. LPS or vehicle once every 2 weeks for 3 months. Animals were sacrificed 3 or 15 days after the last injection, under anesthesia with ketamine hydrochloride (150 mg/Kg) plus medetomidine (0.3 mg/Kg), and transcardially perfused with cold saline. CP isolation was made under conventional light microscopy (SZX7, Olympus, Hamburg, Germany) and tissue was rapidly removed, frozen in dry ice and stored at -80°C. For the microarray experiment three polls of CP (from 3 animals each) were prepared for each experimental group and for each time point. For the qRT-PCR study five pools of CP (from 3 animals each) were used for each experimental group and for each time point.

### Microarray experimental design and data analysis

Total RNA was isolated using Trizol reagent (Invitrogen, Calrsbad, CA, USA). Total RNA quality was assessed using the Agilent Bioanalyzer (Santa Clara, CA, USA). After quality assessment, 100 ng of total RNA from 3 control pools and 3 LPS pools, for each time point, were amplified and labelled with Illumina TotalPrep RNA Amplification Kit according to manufacturer instructions. Each pool was composed of CP collected from 3 animals. The labelled cRNA was then hybridized using Illumina recommended protocol in a total of two Illumina Whole-genome Mouseref-8 expression Beadchips (San Diego, CA, USA). This mouse beadchip contains eight arrays, each comprising a total of 24,000 well-annotated RefSeq transcripts.

After scanning, raw data from BeadStudio software (San Diego, CA, USA) was read into R/Bioconductor. Quality control using inter-array Pearson correlation and clustering based on variance allowed us to ensure that there was reproducibility between the replicates (data not shown). Data was normalized using quantile normalization. A linear model was applied to the normalized data using Limma package in R/Bioconductor [39]. The CP transcriptome of the LPS injected animals was analysed and compared with that of control animals. A contrast analysis was applied and differentially expressed genes were selected using a Bayesian approach with a false discovery rate (FDR) of 5%. The differentially expressed genes were categorized using Gene Ontology from Biomart http://www.biomart.org/ or Ingenuity tools (Redwood City, CA, USA). Enrichment analysis was performed using the DAVID http://david.niaid.nih.gov/david/ease.htm and the Ingenuity software's.

### Gene expression measurements by qRT-PCR

500 ng of total RNA, isolated as described above, were amplified using a SuperScript RNA Amplification System (Invitrogen) according to the manufacturer's instructions. After amplification, RNA was reverse transcribed into first strand cDNA using random hexamers of the superscript first-strand synthesis system for RT-PCR (Invitrogen).

qRT-PCR analysis was used to measure the expression levels of selected mRNA transcripts. Primers were designed using the Primer3 software [40] on the basis of the respective GenBank sequences. The expression level of the reference gene hypoxanthine guanine phosphoribosyl transferase (*Hprt*) (accession number from GenBank: NM_013556) was used as internal standard for normalization. All the other accession numbers and primers sequences are available upon request. Reactions using equal amounts of total RNA from each sample were carried out on a CFX 96™ real-time system instrument (Bio-Rad Laboratories, Hercules, CA, USA) with the QuantiTect SYBR Green RT-PCR reagent kit (Qiagen, Hamburg, Germany) according to the manufacturer's instructions. Product fluorescence was detected at the end of the elongation cycle. All melting curves exhibited a single sharp peak at the expected temperature.

### Statistical analysis

Values are reported as mean ± SEM. Statistical significance was determined using the non-parametric Mann-Whitney test, with differences considered significant at p < 0.05.

## Authors' contributions

JAP, MCN, JCS, NS: conception and design, analysis and data interpretation. FM, JCS: conception and design, acquisition of data and analysis and interpretation of data. GC, DHG: microarray analysis and data interpretation. All authors read and approved the final manuscript.
